# ProteoModlR for functional proteomic analysis

**DOI:** 10.1186/s12859-017-1563-6

**Published:** 2017-03-04

**Authors:** Paolo Cifani, Mojdeh Shakiba, Sagar Chhangawala, Alex Kentsis

**Affiliations:** 1 Molecular Pharmacology Program, Sloan Kettering Institute, Memorial Sloan Kettering Cancer Center, New York, NY USA; 2 Physiology, Biophysics and Systems Biology Program, Weill Cornell Graduate School of Medical Sciences, New York, NY USA; 3 Department of Pediatrics and Memorial Sloan Kettering Cancer Center, Weill Medical College of Cornell University, New York, NY USA

**Keywords:** Mass spectrometry, Quantitative proteomics, Post-translational modification stoichiometry, Functional analysis, R

## Abstract

**Background:**

High-accuracy mass spectrometry enables near comprehensive quantification of the components of the cellular proteomes, increasingly including their chemically modified variants. Likewise, large-scale libraries of quantified synthetic peptides are becoming available, enabling absolute quantification of chemically modified proteoforms, and therefore systems-level analyses of changes of their absolute abundance and stoichiometry. Existing computational methods provide advanced tools for mass spectral analysis and statistical inference, but lack integrated functions for quantitative analysis of post-translationally modified proteins and their modification stoichiometry.

**Results:**

Here, we develop ProteoModlR, a program for quantitative analysis of abundance and stoichiometry of post-translational chemical modifications across temporal and steady-state biological states. While ProteoModlR is intended for the analysis of experiments using isotopically labeled reference peptides for absolute quantitation, it also supports the analysis of labeled and label-free data, acquired in both data-dependent and data-independent modes for relative quantitation. Moreover, ProteoModlR enables functional analysis of sparsely sampled quantitative mass spectrometry experiments by inferring the missing values from the available measurements, without imputation. The implemented architecture includes parsing and normalization functions to control for common sources of technical variation. Finally, ProteoModlR’s modular design and interchangeable format are optimally suited for integration with existing computational proteomics tools, thereby facilitating comprehensive quantitative analysis of cellular signaling.

**Conclusions:**

ProteoModlR and its documentation are available for download at http://github.com/kentsisresearchgroup/ProteoModlR as a stand-alone R package.

**Electronic supplementary material:**

The online version of this article (doi:10.1186/s12859-017-1563-6) contains supplementary material, which is available to authorized users.

## Background

Studies of cellular signaling have historically relied on immunoassays due to their ease of use and widespread accessibility. However, their variable specificity, semi-quantitative nature and availability only for selected proteins and post-translational modifications (PTMs) hinder their application to biological problems that require accurate, precise and multi-parametric measurements. High-resolution mass spectrometry (MS) satisfies these requirements, enabling quantitative measurements of post-translational modifications across thousands of proteins, with proteome coverage approaching genome-scale levels [1–3].

In bottom-up mass spectrometry, proteins are cleaved into peptides, whose identity is determined from the analysis of their fragmentation mass spectra. Likewise, peptide adducts from post-translational chemical modifications produce mass shifts in precursor and fragment ions that are used for PTM identification and localization [4, 5]. Once identification of peptides and PTMs is accomplished, relative peptide abundance can be estimated from the intensity of the corresponding MS signal, i.e. from extracted ion current of either precursor or fragment ions. However, the chemical composition of each peptide determines its ionization properties and therefore its specific MS signal-response function. Absolute peptide quantitation, required for stoichiometry calculations, depends on the use of reference standards to control for variable chromatographic and ionization properties of peptides [6, 7]. Furthermore, apparent differences in MS signals can be due to variations in both peptide abundance and/or its modification stoichiometry. Thus, functional proteomic analyses require deconvolution of these two distinct biological processes, in addition to control of sources of technical variation [8].

Increasing complexity of biological mass spectrometric experiments has prompted the development of several computational programs for mass spectral analysis, ion current extraction, and statistical inference. For example, MaxQuant enables peptide identification, quantitation and PTM site localization [9, 10]. Skyline is designed for precise peptide quantification based on extracted ion chromatograms (XIC) [11]. MSstats permits statistical comparisons of quantitative proteomics data, whereas NetworKIN and Scaffold enable functional annotation [12, 13]. However, these programs lack integrated functions for quantitative analysis of post-translationally modified proteins or do not compute modification stoichiometry.

Here, we describe a generalized method for quantitative analysis of differential abundance and PTM stoichiometry from peptide-based proteomics data. We implemented this approach in an open-source R program [14], named ProteoModlR, which also offers normalization functionalities to improve the analytical accuracy through control of common sources of technical variation. Due to its modular design and interchangeable format, ProteoModlR can process the output of a variety of current proteomic programs, such as MaxQuant and Skyline, and facilitates the use of quantitative proteomics for the analysis of cellular signaling.

## Implementation

ProteoModlR calculates differences in peptide abundance and PTM stoichiometry (i.e. the molar fraction of peptide bearing a given PTM, compared to the total amount of that peptide) across different experimental conditions and biologic states, based on intensity measurements obtained using any program for mass spectrometric analysis. The input dataset requires identifiers for proteins, peptides and modifications, along with the signal intensity measurements for each peptide, formatted as a comma-separated value (CSV) file, that can be generated using programs for quantitative mass spectral analysis such as MaxQuant or Skyline. In particular, Skyline’s ‘Export Report’ can be customized for direct import into ProteoModlR. ProteoModlR also accommodates additional protein and sample annotations, including PTM site information and functional ontology. The currently implemented workflow does not perform protein inference independently and relies instead on the explicitly provided peptide annotation.

**Fig. 1:**
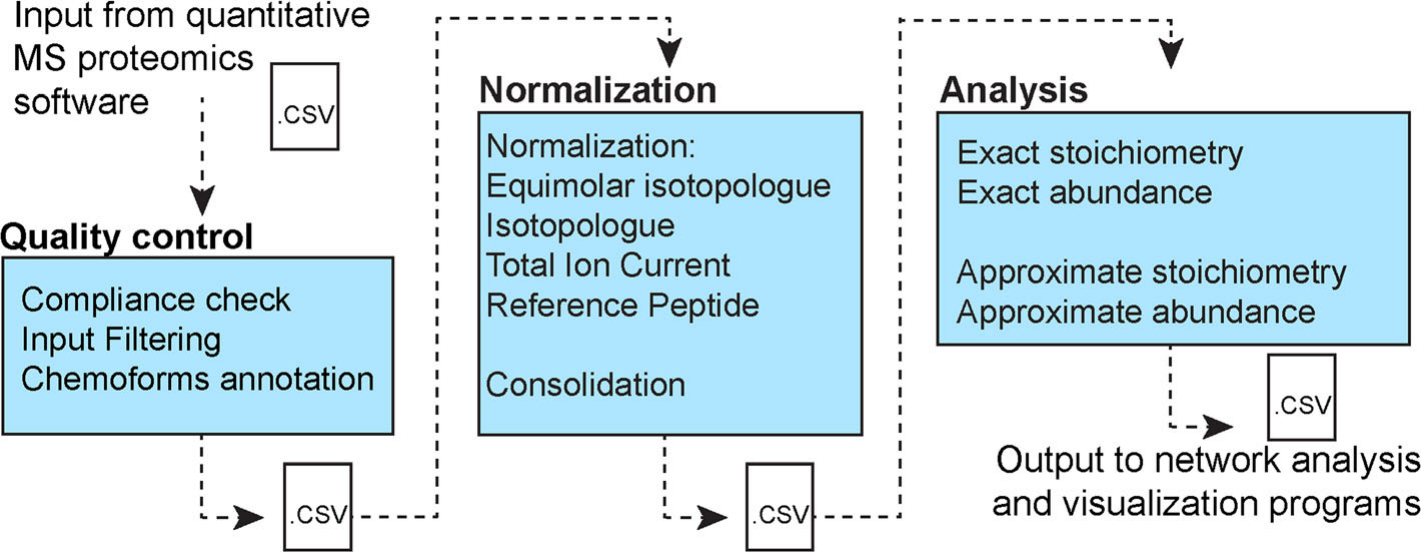
ProteoModlR’s schema. ProteoModlR consists of 3 modules, accepts data from common programs for mass spectral analysis, and generates open-format results that integrate with existing R programs for network analysis and visualization

**Fig. 2:**
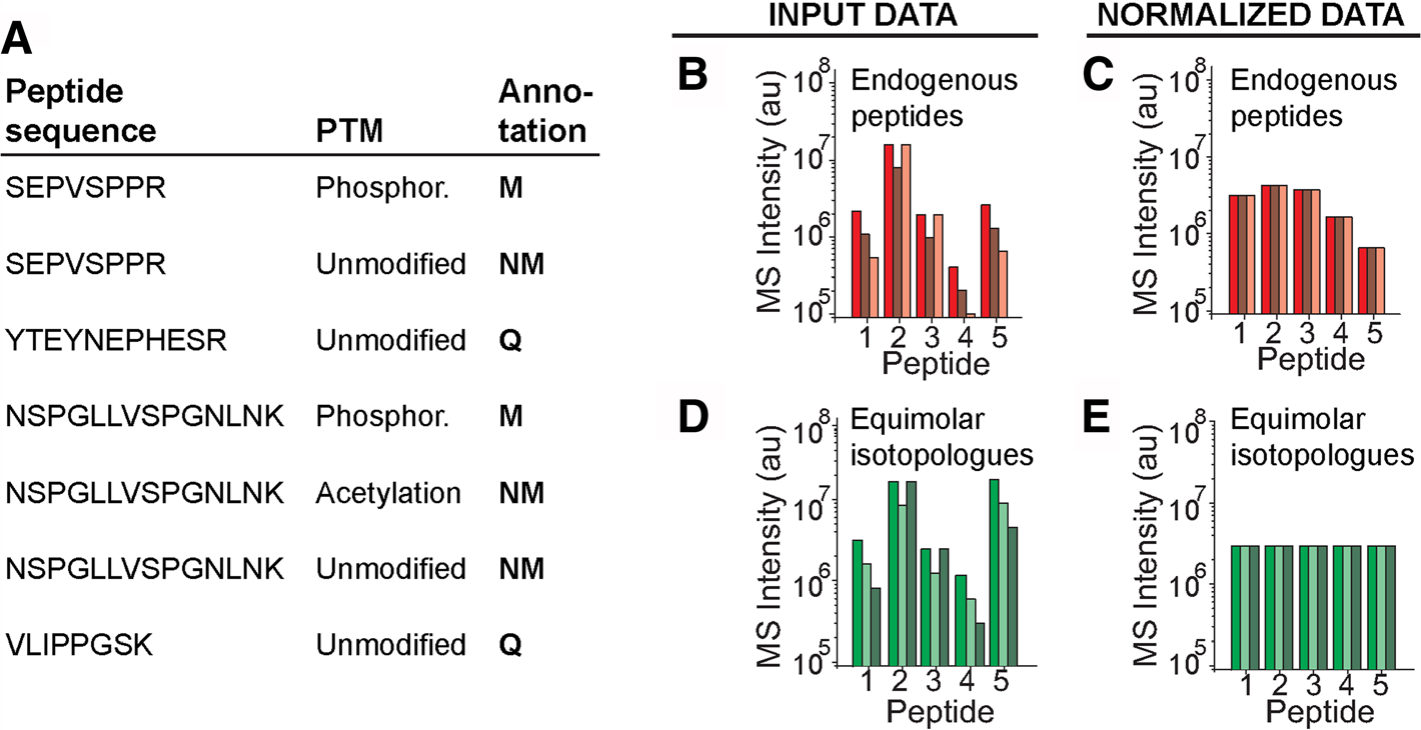
ProteoModlR annotates peptides based on their PTM status and controls potential sources of technical variation. **a** Peptide annotation of peptides from a hypothetical protein depends on available quantified chemoforms. **b** Quantitation across three replicate measurements (shades of red) of five peptides from a protein. **c** ProteoModlR corrects errors introduced by differential ionization efficiency and technical variability, equalizing the signal recorded for equimolar reference isotopologues (**d**–**e**)

ProteoModlR consists of 3 modules performing *Quality Control*, *Normalization*, and *Analysis* (Fig. 1, Additional file 1: Figure S1). For each protein in the input, the *Quality Control* module parses the available peptides based on their modification status. For a given peptide sequence, if none of the chemoforms bears the modification of interest, then the peptide is used for protein quantification (therefore labeled as ‘Q’). If, on the other hand, one or more chemoforms bears the modification of interest, then all peptides are labeled as ‘modified’ (M) or ‘not modified’ (NM), depending on their annotated PTM status (Fig. 2a). Furthermore, *Quality Control* checks the correct formatting of the input file, and removes proteins with no quantified peptides. The current implementation of *Quality Control* is based on exact amino acid sequences: if incomplete or nonspecific proteolysis results in peptides with different termini, each of them is considered separately. After annotation, the dataset is exported as a CSV file.

Variable ionization efficiency prevents direct conversion of MS signal intensities into absolute peptide abundance, which is required for calculating PTM stoichiometries. The relative abundance and stoichiometry calculations performed by ProteoModlR are therefore based on three assumptions. First, ProteoModlR assumes that the samples contain synthetic isotope-labeled peptides as reference standards, present at equimolar concentration. Second, it assumes that these peptides have linear signal-response functions with slopes equal or close to one. In practice, this requires confirmation, as is currently routine for absolute quantitation methods [6, 15–17]. Third, ProteoModlR assumes that all or most of the variants of each peptide produced by chemical modification of a given protein (hereafter named ‘chemoforms’) are quantified. This implies that the total molarity of all peptide chemoforms is equal to that of the unmodified peptide, and to that of the protein from which they originated.

For datasets that meet the aforementioned assumptions, the *Normalization* module normalizes the intensity values from the *Quality Control* output, equalizing the intensity of the equimolar reference peptides. This normalization (termed *equimolar isotopologue* normalization) can also correct for possible experimental alterations to chemoforms molarity, such as those produced by enrichment of chemically modified peptides [18], or due to variable recovery from chromatographic separation. This module also reduces technical variability produced by other sources, such as uneven sample loading and variable efficiency of electro-spray ionization (Fig. 2b, Additional file 2: Figure S2).

For datasets that may not meet the aforementioned assumptions, ProteoModlR offers three additional normalization modes that can be leveraged for correction of potential technical artifacts. First, normalization by *isotopologue* can be performed if a non-equimolar set of labeled peptides is used, as could be the case for SILAC-labeled proteomes (Additional file 3: Figure S3). Second, normalization by *total ion current* (TIC) can be used, if no isotopically encoded standard is available (Additional file 4: Figure S4). Finally, normalization using a set of *internal reference* peptides can be used to correct for variations in the total protein content per cell (Additional file 5: Figure S5). Recommended normalization strategies for common experimental designs are provided in the Software Documentation (Additional file 6). To permit modular analyses, the output of the *Normalization* module is also exported as a CSV file.

Finally, the *Analysis* module of ProteoModlR calculates differential abundance and PTM stoichiometry based on the annotation performed in *Quality Control*. Relative abundance is calculated using the intensities of ‘Quantification’ peptides, and expressed as the ratio of the intensities in every sample to that of the reference sample. If positional information is provided, PTM stoichiometry is calculated for each site individually, as the ratio between the intensity of the modified peptide over the sum of the intensities of all chemoforms of that peptide. Assuming that the experimental dataset contains quantification annotation for all classes of peptides, as described above, these calculations are referred to as *exact* within ProteoModlR.

**Fig. 3:**
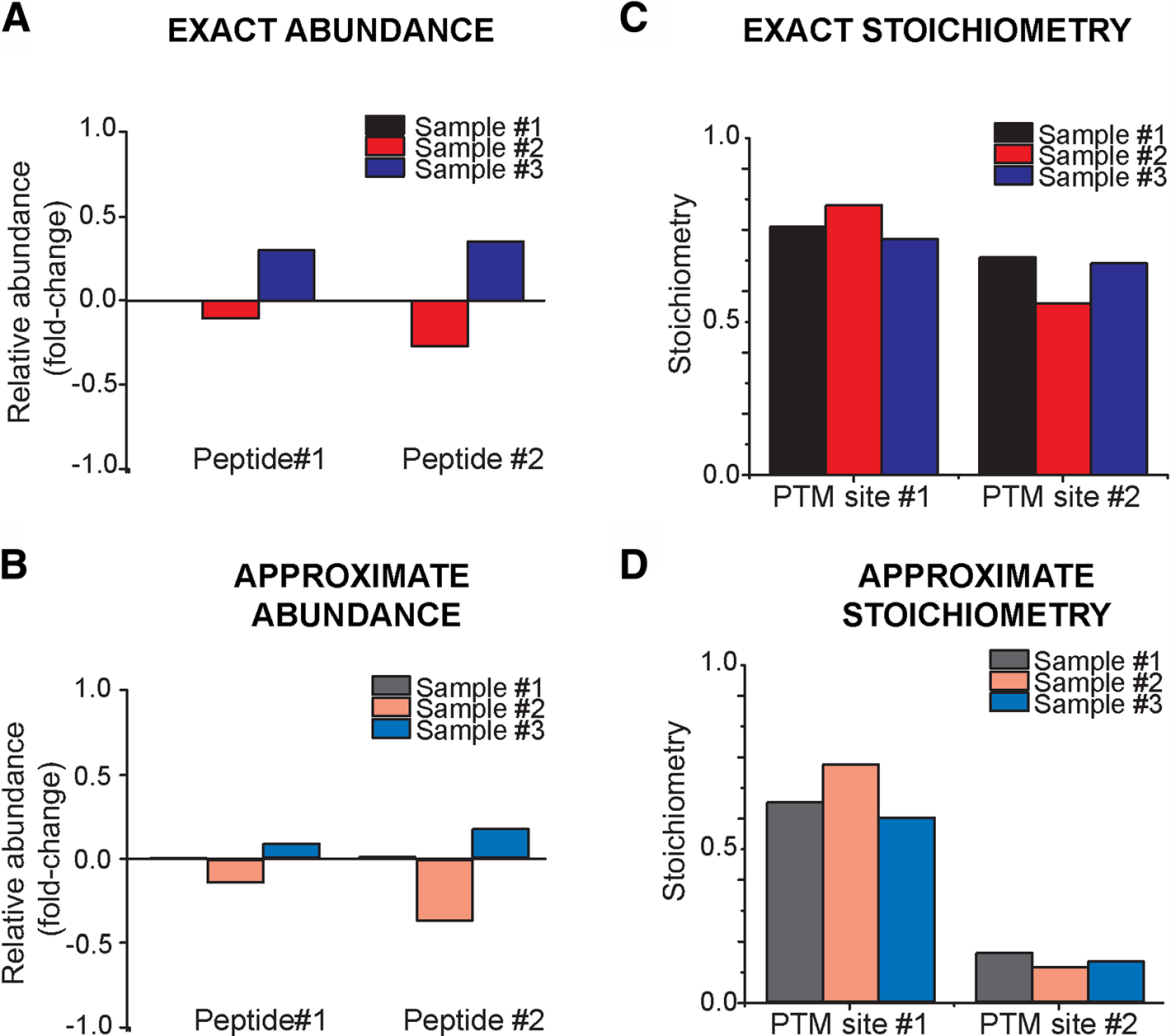
ProteoModlR computes exact and approximate abundance and PTM stoichiometry based on the available set of peptides for a given protein. Exact and approximate calculations for abundance and stoichiometry were tested on simulated datasets modeling a hypothetical protein producing four peptides, two of which bear a PTM. If the input file contains all ‘Q’, ‘M’ and ‘NM’ peptides, then ProteoModlR computes ‘exact’ relative peptide abundance, expressed as fold change compared to Sample #1 (**a**) and stoichiometry (**c**) If ‘Quantification’ (‘Q’) or non-modified (‘NM’) peptides are not available, ProteoModlR can calculate ‘approximate’ relative abundances (**b**) and 'approximate stoichiometry (**d**) resembling the exact values (Pearson product–moment correlation coefficient = 0.98)

To enable approximate analysis of experimental data that are incompletely annotated, as in the case of proteins lacking quantified ‘Q’ or ‘NM’ peptides, ProteoModlR can be used to infer the missing values based on the intensities of the detected peptides (Fig. 3). As described above, ProteoModlR assumes that the total molarity of all chemoforms of a peptide is equal to that of the unmodified ‘Q’ peptide. If ‘Q’ peptides are not detected, then *approximate* abundance can be estimated using the sum of the intensity of all chemoforms of modified peptides (‘M’ and ‘NM’ peptides). Likewise, *approximate* PTM stoichiometry can be estimated using the intensity of unrelated ‘Q’ peptide as a proxy for total protein abundance, instead of the total intensity of all chemoforms for that peptide (Additional file 7: Figure S6). When no equimolar isotopologue standard is used, the results of these calculations approximate biologic differences. In the case of affinity enrichment of chemically modified peptides, such as in the case of conventional phosphoproteomic measurements, ProteoModlR requires appropriate internal standards to ensure accuracy of the results. The output of the *Analysis* module is exported as a CSV file, allowing for subsequent statistical and network analysis using existing programs, such as those implemented in Bioconductor. Further details of the operations performed by each module are provided in the Software Documentation (Additional file 6 and available at https://github.com/kentsisresearchgroup/ProteoModlR).

## Results and discussion

To test the functionalities of ProteoModlR, first we used simulated datasets, modeling MS measurements of peptides generated from a hypothetical phosphorylated protein and from loading controls, across three biological samples (Additional file 8: Simulated Dataset 1 and Additional file 9: Simulated Dataset 7). The datasets used to test the performance of *Normalization* module simulated errors within identical samples, introduced by common sources of technical variation: i) deterioration of the efficiency of the LC-ESI-MS instrumentation; ii) variable sample loading from inaccurate estimation of protein content, and iii) variable sample loading from significant variation of specific proteins per cell. In each case, we were able to correct these errors by using ProteoModlR to apply normalization by isotopologue, equimolar isotopologue, total ion current, or internal reference peptides (Additional files 2, 3, 4, 5: Figures S2-S5, Additional files 10, 11, 12, 13, 14: Simulated Datasets 2–6).

For data containing complete measurements of all peptides under all conditions, we used ProteoModlR to calculate exact peptide abundances and PTM stoichiometries (Fig. 3a, c). Simulating different types of incomplete datasets, we then used ProteoModlR to approximate peptide abundance and PTM stoichiometries (Figs. 3b, d, Additional file 7: Figure S6, Additional files 9, 15, 16, 17, 18: Simulated Dataset 7–11). Similar results were obtained for the comparison between exact and approximate PTM stoichiometries. As such, approximate calculations generated results that highly correlated with the expected values (Pearson product–moment correlation coefficient of 0.98 and 0.95 for abundance and stoichiometry, respectively). Thus, this approximation may suffice for semi-quantitative studies, when complete data are not available.

To test ProteoModlR’s performance for the analysis of large-scale quantitative mass spectrometry data and compatibility with existing programs of mass spectral analysis, we analyzed changes in relative protein abundance and phosphorylation stoichiometries of CD8+ T cells upon interleukin-2 (IL-2) stimulation, as measured using SILAC and MaxQuant (PRIDE accession: PXD004645) [19]. First, we used ProteoModlR’s *Quality Control* and *Normalization* modules to correct for differences in loading between steady-state (light isotope) and stimulated (heavy isotope) conditions using the total ion current normalization routine. Since these experiments were performed using metal affinity chromatography, detection was biased against ‘Q’ and ‘NM’ peptides, and ‘M’ phosphopeptides accounted for 84% of the detected chemoforms. We thus used the *approximate* abundance and stoichiometry calculation routines in the *Analysis* module to complement *exact* calculations.

**Fig. 4:**
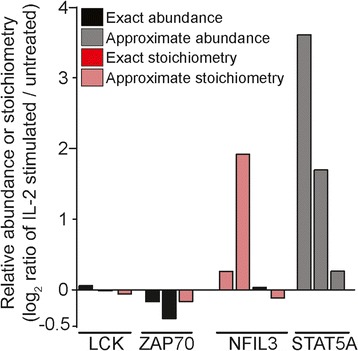
ProteoModlR enables large-scale analysis of experimentally measured protein abundance and PTM stoichiometry. Relative abundance of four representative proteins from 2784 proteins analyzed from SILAC-labeled CD8+ T cells stimulated with IL-2, as compared to unstimulated control, and analyzed using MaxQuant [19]. T-cell receptor signaling-dependent LCK and ZAP70 proteins exhibit no changes upon IL-2 stimulation, whereas IL-2 receptor dependent STAT5A and NFIL3 exhibits increases in abundance or phosphorylation stoichiometry upon IL-2 stimulation

Using this approach, we used ProteoModlR to calculate specific changes in both abundance and phosphorylation stoichiometry of 2794 proteins upon IL-2 stimulation (Fig. 4, Additional file 19: Table S1). For example, we found no changes in abundance or phosphorylation stoichiometry of LCK and ZAP70, in agreement with their known functions in T cell receptor (TCR) but not IL-2 receptor signaling [19]. In contrast, we observed apparent increases in abundance and phosphorylation stoichiometry of STAT5A and NFIL3, in agreement with their expected involvement in JAK1/2-dependent signaling induced by IL-2 receptor stimulation [19]. The apparent increase in STAT5A abundance without a change in phosphorylation stoichiometry may be due to an increase in STAT5A phosphorylation and/or increased protein abundance. Thus, ProteoModlR enabled both exact and approximate large-scale calculations of protein abundance and phosphorylation stoichiometry, depending on the presence of their chemoforms.

Functional analysis of biological processes requires precise characterization of the activation status of the relevant effector proteins. In this context, cellular protein abundance and post-translational modification have important biological functions. Large libraries of synthetic peptides now enable near-comprehensive MS analysis of peptide chemoforms and deconvolution of their respective ionization efficiency. Consequently, it is now becoming feasible to calculate the modification stoichiometry of large sets of proteoforms, which is important for functional analysis of cellular signaling [8, 20, 21]. However, while existing mass spectrometry analysis programs allow extraction and visualization of protein expression levels from quantitative mass spectrometry experiments, calculations of PTM stoichiometry still require specialized approaches.

ProteoModlR facilitates the analysis of quantitative mass spectrometry experiments by calculating differential protein abundance and PTM stoichiometry across temporal and steady-state biological states. The software deconvolutes the contribution of chemical modifications of peptides to their mass spectrometric signal intensity, thereby calculating both PTM stoichiometry and relative protein abundance. To this end, ProteoModlR annotates the available quantified peptides according to their PTM status, determining for each modification the chemoforms relevant for stoichiometry and abundance calculations. The software integrates normalization functions to correct, based on the signal of synthetic reference standards, MS intensity distortion produced by variable peptide ionization efficiency, as well as other common sources of technical variability. Finally, ProteoModlR calculates relative differences in protein abundance and PTM stoichiometry, thus facilitating analyses of cellular protein function.

Missing values in sparsely annotated datasets are commonly either filtered out or imputed to enable subsequent statistical analysis and functional pathway modeling. ProteoModlR introduces an alternative strategy, based on inferring the quantity of non-detected peptides from the normalized measured intensities of other peptides derived from the same protein. It can thus complement and improve the comprehensiveness of currently available tools for functional analysis. In addition, ProteoModlR’s modular design and flexible workflow allow for its integration with existing proteomics software such as MaxQuant and Skyline, as well as existing statistical and visualization tools available in Bioconductor. Thus, ProteoModlR’s computational framework will prove useful for a wide variety of quantitative mass spectrometry studies, including the comprehensive investigation and quantitative modeling of cellular signaling and biochemical pathways.

## Conclusions

Here we introduce ProteoModlR for quantitative mass spectrometry analysis of post-translationally modified peptides and proteins for functional proteomics of cell signaling.

## Additional files

Additional file 1: Figure S1. Conceptual overview of the operations performed by ProteoModlR. (A) A set of proteoforms is digested into peptides and (B) mixed with an equimolar set of synthetic reference peptides (in blue). (C) MS signal-response is affected by differential ionization efficiency. Furthermore, MS quantification may present missing values. (D) ProteoModlR first annotates the available set of peptides, then (E) corrects errors introduced by technical and biological variability. Finally, (F) exact or approximate calculations are deployed to obtain PTM stoichiometry and abundance. (TIF 919 kb)
Additional file 2: Figure S2. Equimolar Isotopologue normalization corrects for technical variability across measurements, as demonstrated on simulated data. A) Quantitation across three replicate measurements of five peptides from a protein of interest (shades of red) and four peptides from reference proteins (shades of blue). (B) ProteoModlR corrects errors introduced by technical and biological variability. (C) Quantitation of heavy labeled equimolar standard peptides is affected by differential ionization efficiency and technical variability. (D) ProteoModlR equalizes the intensities of the standard isotopologues for each peptide independently. (TIF 9310 kb)
Additional file 3: Figure S3. Isotopologue normalization corrects for technical variability across measurements, as demonstrated on simulated data. (A) Quantitation across three replicate measurements of five peptides from a protein of interest (shades of red) and four peptides from reference proteins (shades of blue). (B) ProteoModlR corrects errors introduced by technical and biological variability. (C) Quantitation of heavy labeled standard peptides is also affected by technical variability. (D) If isotopologue normalization is chosen, ProteoModlR equalizes the intensities of the standard isotopologues for each peptide independently. (TIF 9821 kb)
Additional file 4: Figure S4. Total ion current normalization corrects for technical variability across measurements in absence of isotopically encoded standards, as demonstrated on simulated data. (A) Quantitation across three replicate measurements of five peptides from a protein of interest (shades of red) and four peptides from reference proteins (shades of blue). (B) ProteoModlR corrects errors introduced by technical and biological variability. (C) Total ion current is also affected by technical variability. (D) If total ion current normalization is chosen, ProteoModlR equalizes the sum of the intensities of all peptides in each sample. (TIF 9017 kb)
Additional file 5: Figure S5. Reference peptide normalization corrects for variations in the total protein content per cell across measurements, as demonstrated on simulated data. (A) Quantitation across three replicate measurements of five peptides from a protein of interest (shades of red) and four peptides from reference proteins (shades of blue). (B) ProteoModlR corrects errors introduced by biological factors that vary the total amount of protein per cell, equalizing the intensities of one or more peptides chosen as internal reference. (TIF 4660 kb)
Additional file 6: Software documentation. The document contains detailed description of ProteoModlR implementation as well as user instructions. (PDF 196 kb)
Additional file 7: Figure S6. Output of exact (A) and approximate (B-D) calculations from simulated datasets. The input contained quantitation across three replicate measurements of four peptides, two of which phosphorylated. (TIF 25386 kb)
Additional file 8: Simulated Dataset 1. Normalization simulated datasets – description. Detailed description of the simulated datasets used to test ProteoModlR’s normalization module. (TXT 1 kb)
Additional file 9: Simulated Dataset 7. Analysis simulated datasets – description. Detailed description of the simulated datasets used to test the analysis module ProteoModlR .(TXT 783 bytes)
Additional file 10: Simulated Dataset 2. Normalization_dataset_no_error. The file contains a simulated datasets with identical intensity values for all peptides in all conditions. This dataset is provided as a reference to evaluate the accuracy of normalization strategies. (CSV 4 kb)
Additional file 11: Simulated Dataset 3. Normalization simulated dataset 1a. The file contains the simulated datasets used to model differences in peptide intensity introduced by uneven performance of the LC/MS instrumentation or unequal sample amount. The output shows the result of equimolar isotopologue normalization, as displayed in Additional file 2: Figure S2. (CSV 4 kb)
Additional file 12: Simulated Dataset 4. Normalization simulated dataset 1b. The file contains the simulated datasets used to model differences in peptide intensity introduced by uneven performance of the LC/MS instrumentation or unequal sample amount. The output shows the result of isotopologue normalization, as displayed in Additional file 3: Figure S3. (CSV 4 kb)
Additional file 13: Simulated Dataset 5. Normalization simulated dataset 2. The file contains the simulated datasets used to model differences in peptide intensity introduced by uneven performance of the LC/MS instrumentation or unequal sample amount, with no isotopically labelled internal standard. The output shows the result of normalization by total ion current, as displayed in Additional file 4: Figure S4. (CSV 2 kb)
Additional file 14: Simulated Dataset 6. Normalization simulated dataset 3. The file contains the simulated datasets used to model differences in the peptide intensity introduced by biological factors that vary the protein amount per cell by affecting the expression of a limited subset of the proteome. The output shows the result of isotopologue normalization followed by normalization by reference peptide, as displayed in Additional file 5: Figure S5. (CSV 5 kb)
Additional file 15: Simulated Dataset 8. Analysis simulated dataset 1. This simulated dataset models an input with complete annotation of peptides required for exact calculations in the Analysis module of ProteoModlR. This file provides a reference for approximate calculations, and demonstrates the annotation performed by the quality control module of ProteModlR. (CSV 5 kb)
Additional file 16: Simulated Dataset 9. Analysis simulated dataset 2. This simulated dataset models an input with incomplete annotation of peptides required for exact calculation of abundance in the Analysis module of ProteoModlR. (CSV 5 kb)
Additional file 17: Simulated Dataset 10. Analysis simulated dataset 3. This simulated dataset models an input with incomplete annotation of chemically modified peptides intensity, required for exact calculation of stoichiometry in the Analysis module of ProteoModlR. (CSV 5 kb)
Additional file 18: Simulated Dataset 11. Analysis simulated dataset 4. This simulated dataset models an input with incomplete annotation of non-chemically modified peptides intensity, required for exact calculation of stoichiometry in the Analysis module of ProteoModlR. (CSV 5 kb)
Additional file 19: Table S1. Chemoforms available for abundance and stoichiometry calculations from experimentally derived data. The table contains the peptides available in the experimentally derived dataset (Fig. 4, [19]) for protein LCK, ZAP70, NFIL3 and STAT5A. For each chemoform, modification status and *Quality Control* annotation is reported. (TIF 4000 kb)

## Abbreviations

DDA: Data-dependent acquisition
ESI: Electro-spray ionization
MS: Mass spectrometry
PTM: Post-translational modification
SILAC: Stable isotope labeling by amino acids in cell culture
XIC: Extracted ion chromatogram
