# Supplementary figures and images for: ProteoModlR for functional proteomic analysis
Source: BMC Bioinformatics. 2017 Mar 4;18:153. doi: 10.1186/s12859-017-1563-6 (PMC5336658; doi:10.1186/s12859-017-1563-6)

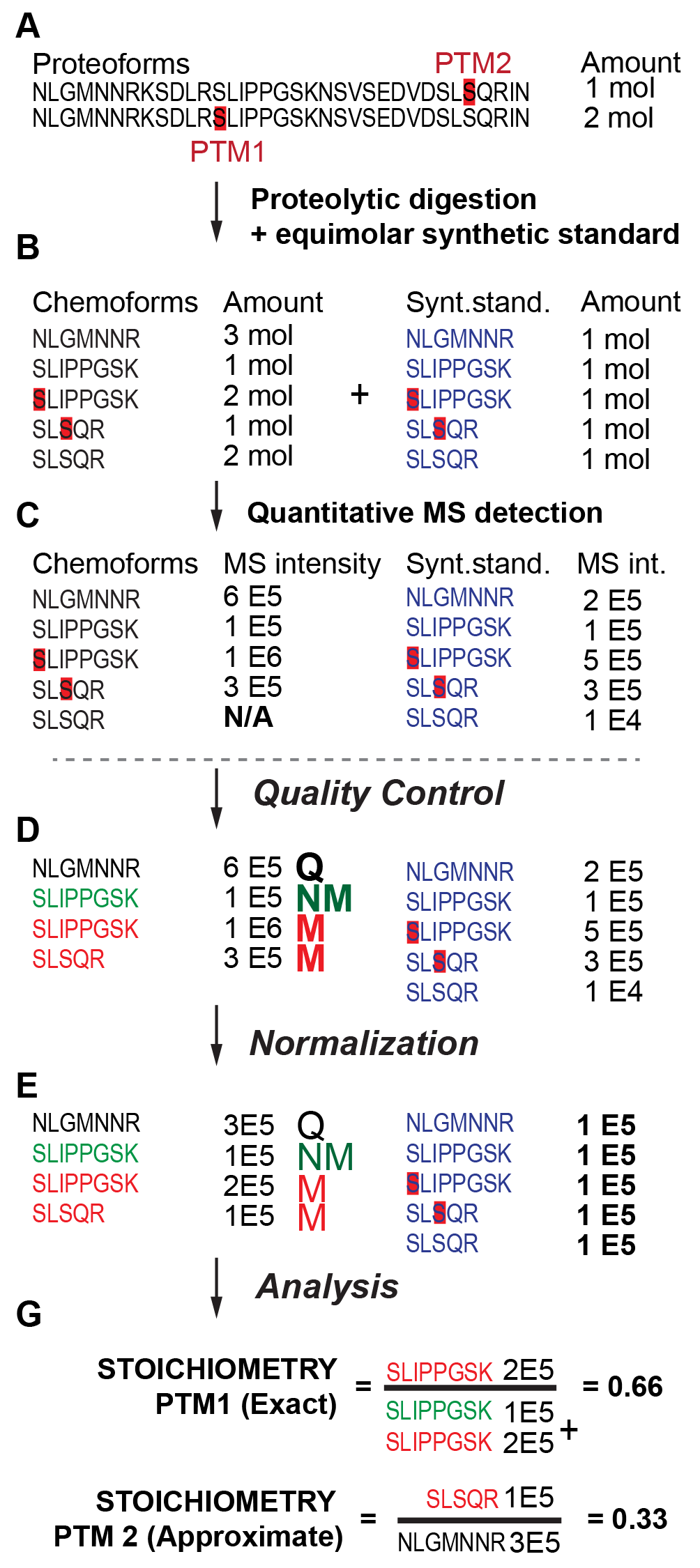

Supplement: Additional file 1: Figure S1. — Conceptual overview of the operations performed by ProteoModlR. (A) A set of proteoforms is digested into peptides and (B) mixed with an equimolar set of synthetic reference peptides (in blue). (C) MS signal-response is affected by differential ionization efficiency. Furthermore, MS quantification may present missing values. (D) ProteoModlR first annotates the available set of peptides, then (E) corrects errors introduced by technical and biological variability. Finally, (F) exact or approximate calculations are deployed to obtain PTM stoichiometry and abundance. (TIF 919 kb) [file 12859_2017_1563_MOESM1_ESM.tif]

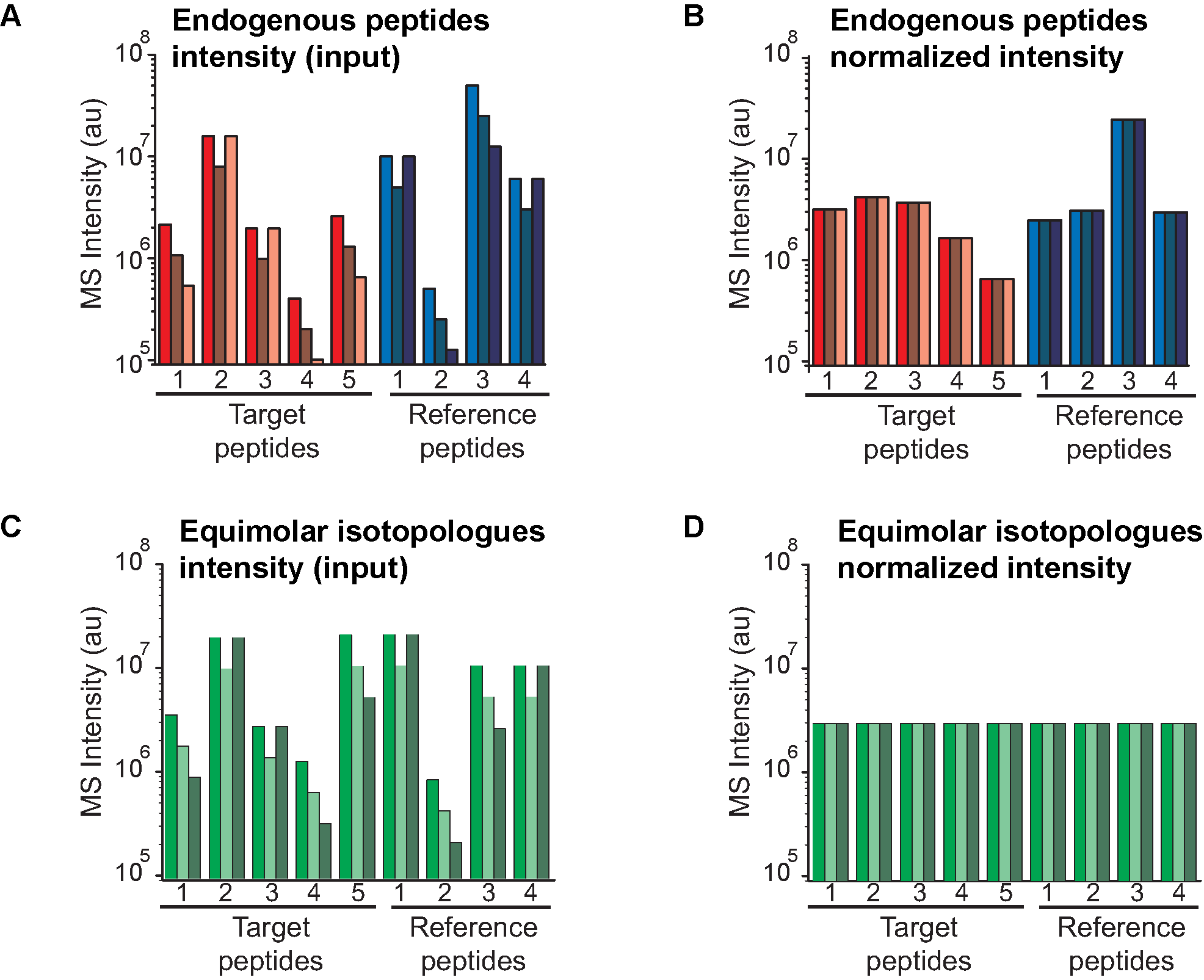

Supplement: Additional file 2: Figure S2. — Equimolar Isotopologue normalization corrects for technical variability across measurements, as demonstrated on simulated data. A) Quantitation across three replicate measurements of five peptides from a protein of interest (shades of red) and four peptides from reference proteins (shades of blue). (B) ProteoModlR corrects errors introduced by technical and biological variability. (C) Quantitation of heavy labeled equimolar standard peptides is affected by differential ionization efficiency and technical variability. (D) ProteoModlR equalizes the intensities of the standard isotopologues for each peptide independently. (TIF 9310 kb) [file 12859_2017_1563_MOESM2_ESM.tif]

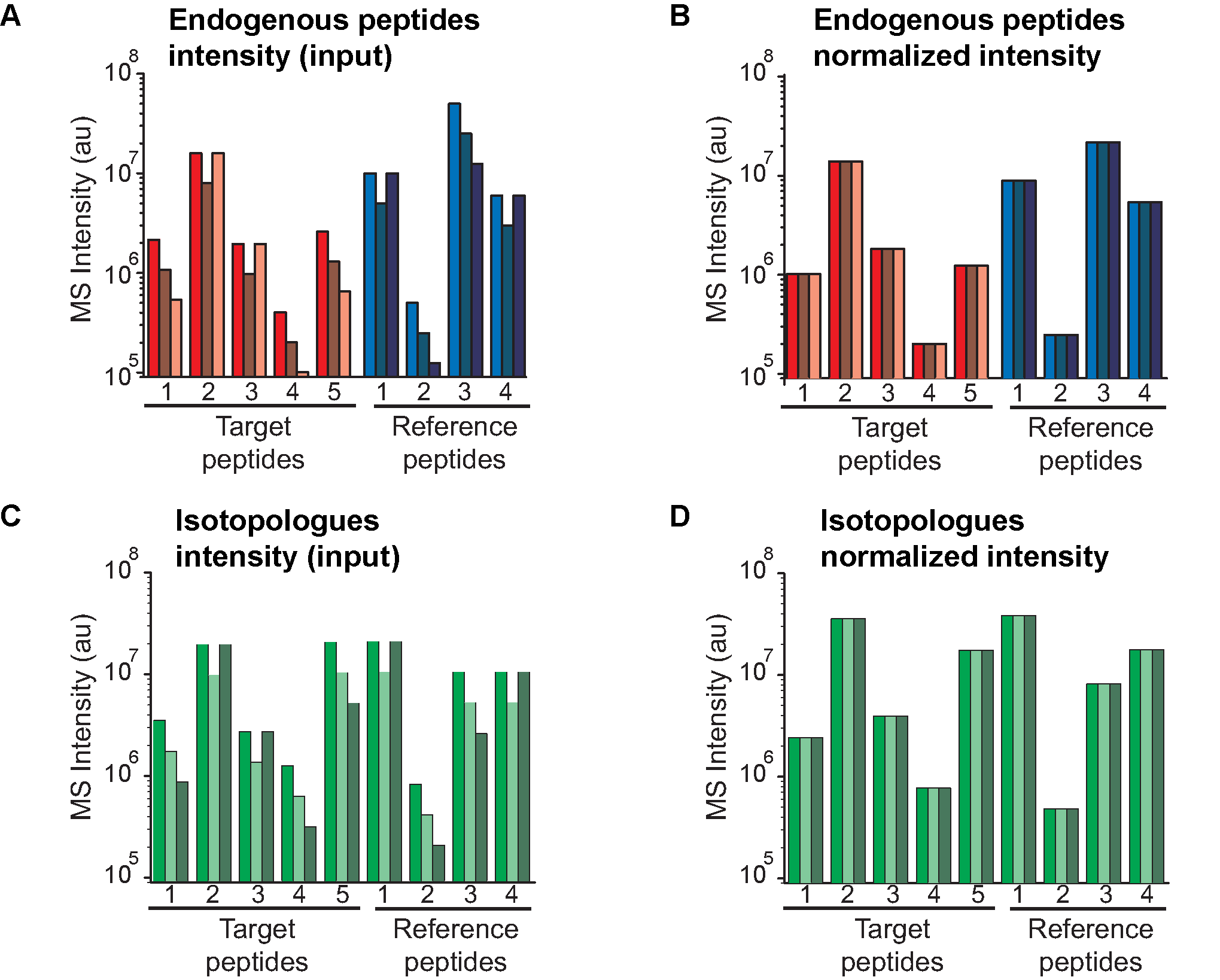

Supplement: Additional file 3: Figure S3. — Isotopologue normalization corrects for technical variability across measurements, as demonstrated on simulated data. (A) Quantitation across three replicate measurements of five peptides from a protein of interest (shades of red) and four peptides from reference proteins (shades of blue). (B) ProteoModlR corrects errors introduced by technical and biological variability. (C) Quantitation of heavy labeled standard peptides is also affected by technical variability. (D) If isotopologue normalization is chosen, ProteoModlR equalizes the intensities of the standard isotopologues for each peptide independently. (TIF 9821 kb) [file 12859_2017_1563_MOESM3_ESM.tif]

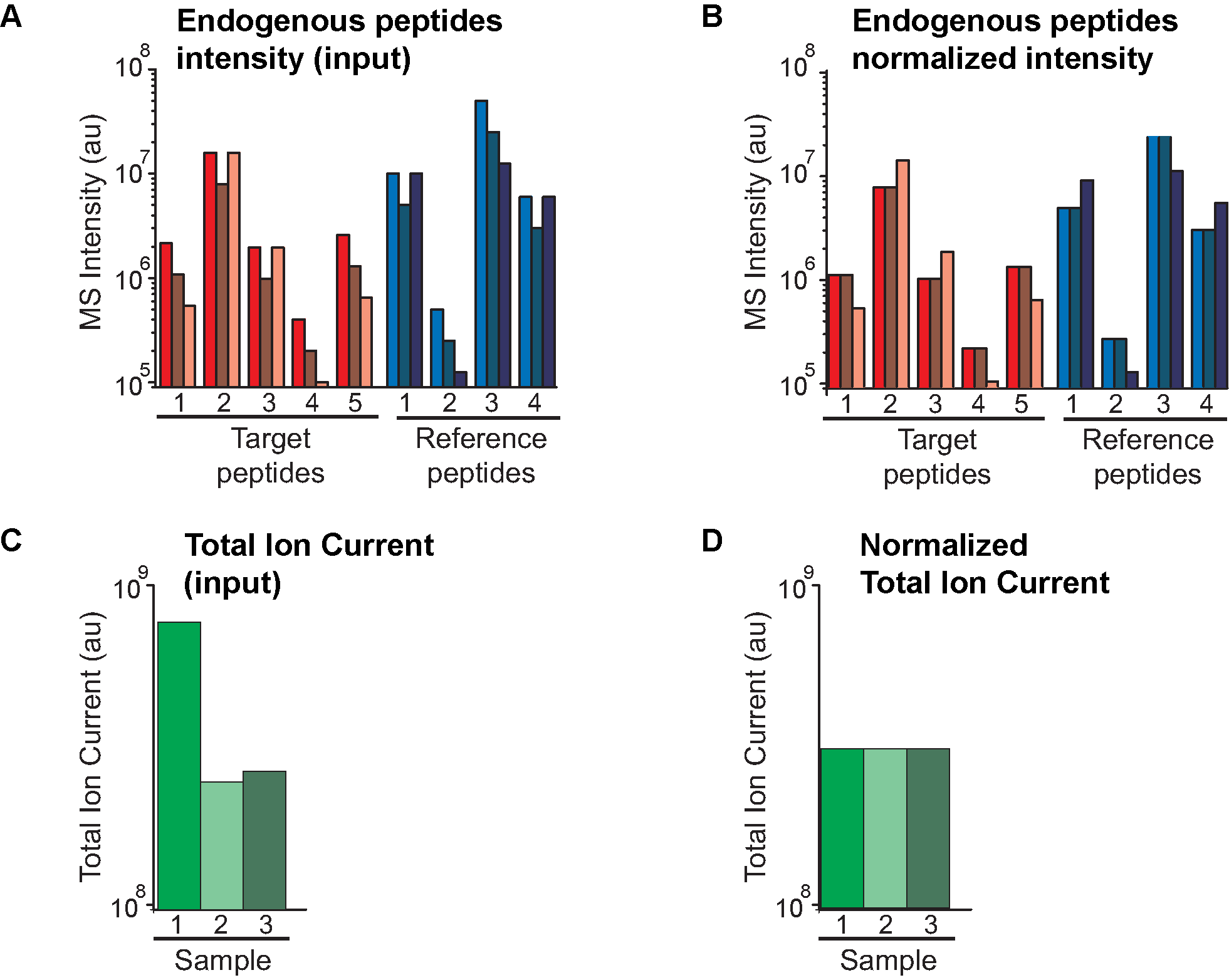

Supplement: Additional file 4: Figure S4. — Total ion current normalization corrects for technical variability across measurements in absence of isotopically encoded standards, as demonstrated on simulated data. (A) Quantitation across three replicate measurements of five peptides from a protein of interest (shades of red) and four peptides from reference proteins (shades of blue). (B) ProteoModlR corrects errors introduced by technical and biological variability. (C) Total ion current is also affected by technical variability. (D) If total ion current normalization is chosen, ProteoModlR equalizes the sum of the intensities of all peptides in each sample. (TIF 9017 kb) [file 12859_2017_1563_MOESM4_ESM.tif]

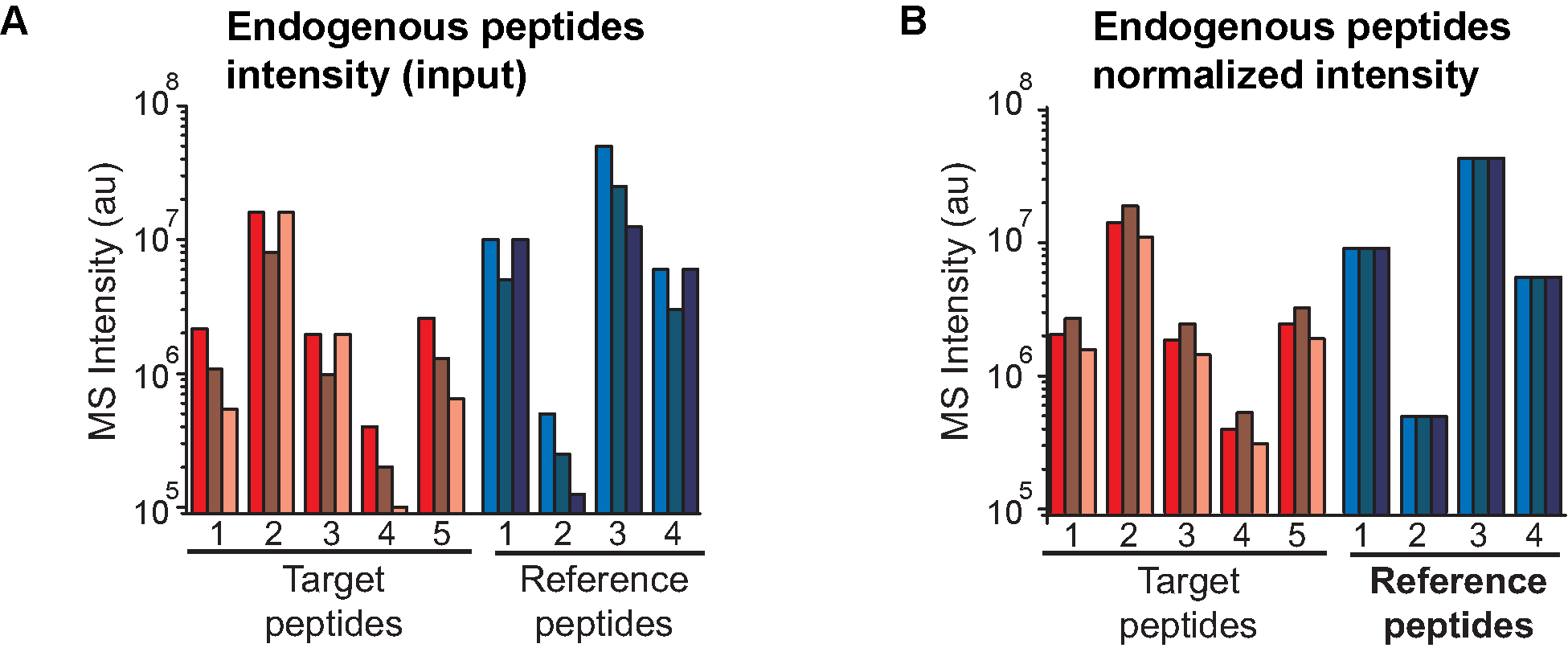

Supplement: Additional file 5: Figure S5. — Reference peptide normalization corrects for variations in the total protein content per cell across measurements, as demonstrated on simulated data. (A) Quantitation across three replicate measurements of five peptides from a protein of interest (shades of red) and four peptides from reference proteins (shades of blue). (B) ProteoModlR corrects errors introduced by biological factors that vary the total amount of protein per cell, equalizing the intensities of one or more peptides chosen as internal reference. (TIF 4660 kb) [file 12859_2017_1563_MOESM5_ESM.tif]

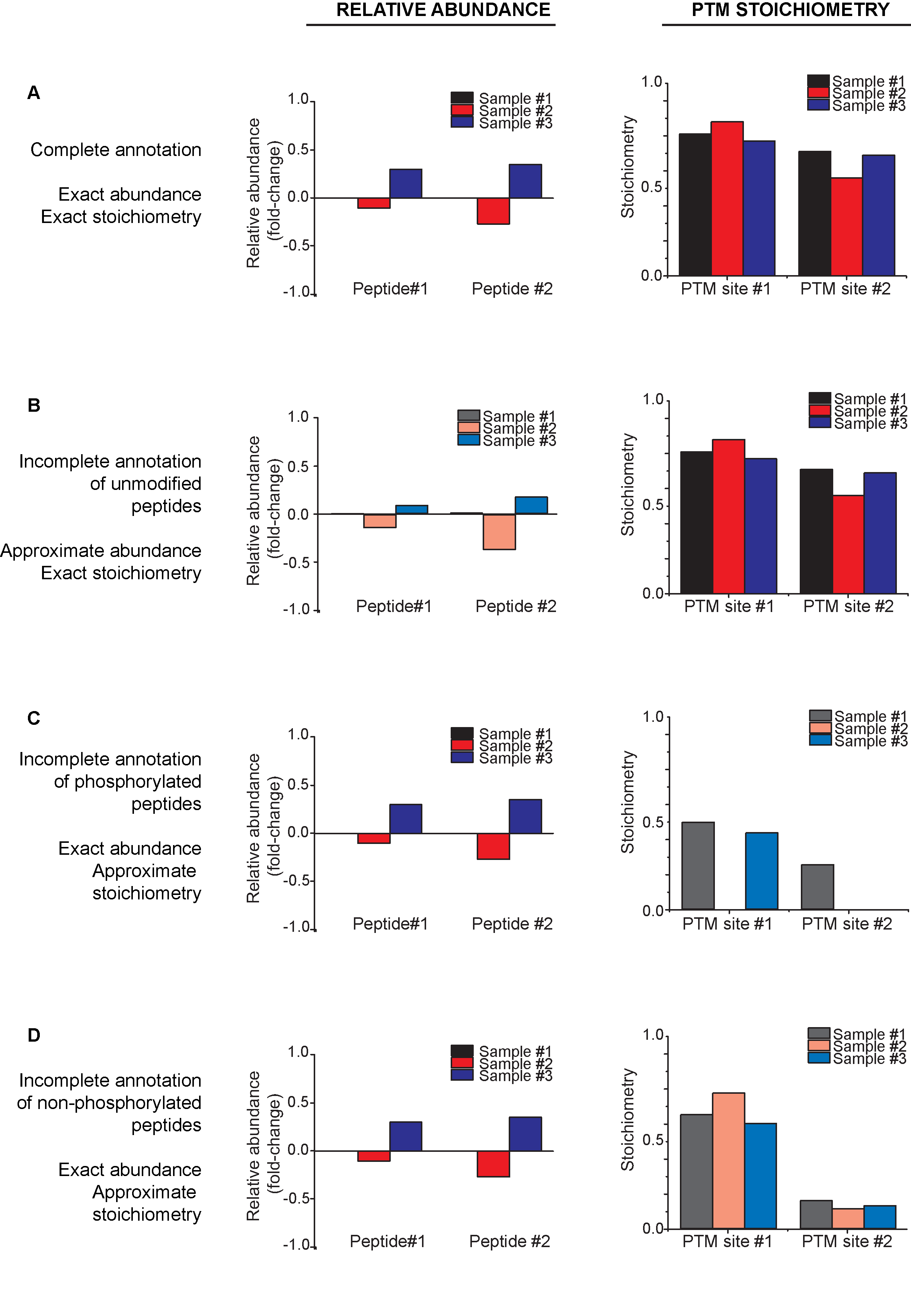

Supplement: Additional file 7: Figure S6. — Output of exact (A) and approximate (B-D) calculations from simulated datasets. The input contained quantitation across three replicate measurements of four peptides, two of which phosphorylated. (TIF 25386 kb) [file 12859_2017_1563_MOESM7_ESM.tif]

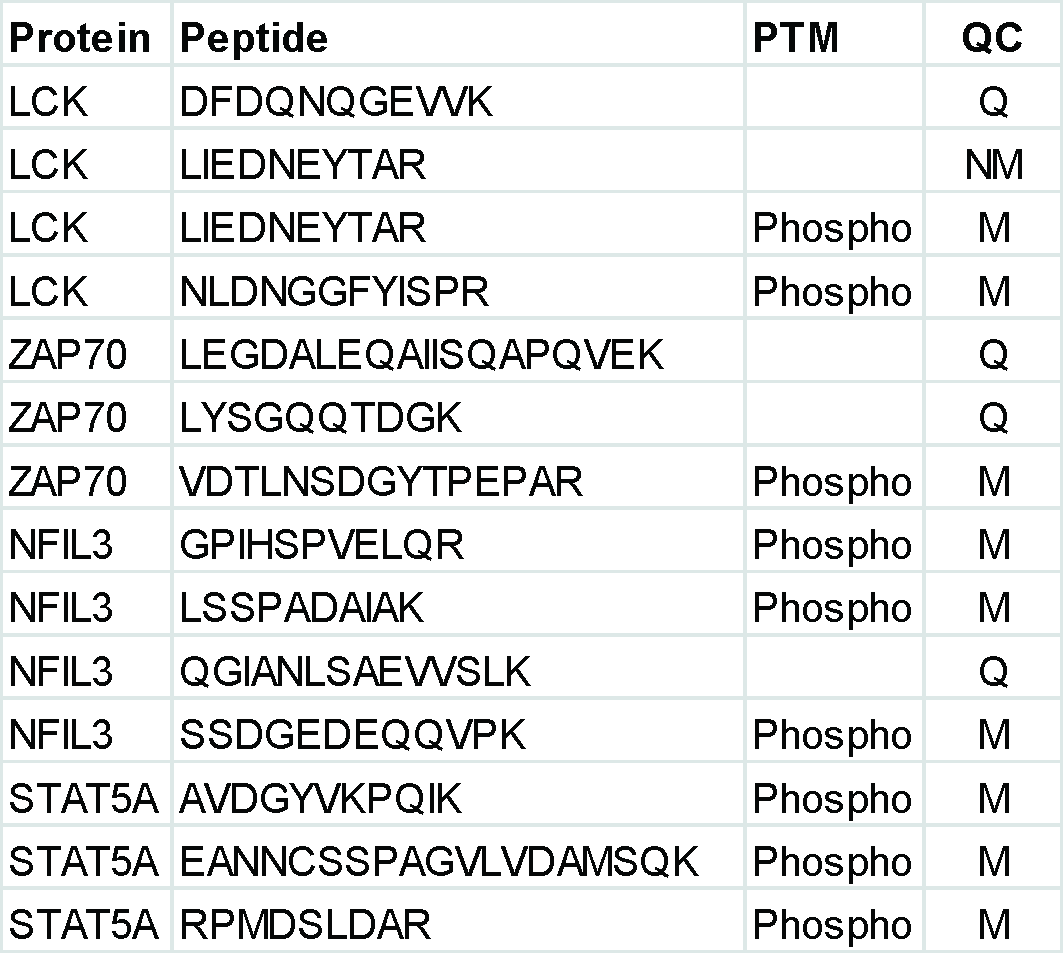

Supplement: Additional file 19: Table S1. — Chemoforms available for abundance and stoichiometry calculations from experimentally derived data. The table contains the peptides available in the experimentally derived dataset (Fig. 4, [19]) for protein LCK, ZAP70, NFIL3 and STAT5A. For each chemoform, modification status and Quality Control annotation is reported. (TIF 4000 kb) [file 12859_2017_1563_MOESM19_ESM.tif]
