# Supplementary material for: ProteoModlR for functional proteomic analysis
Source: BMC Bioinformatics. 2017 Mar 4;18:153. doi: 10.1186/s12859-017-1563-6 (PMC5336658; doi:10.1186/s12859-017-1563-6)
Supplement: Additional file 6: Software documentation. — The document contains detailed description of ProteoModlR implementation as well as user instructions. (PDF 196 kb) [file 12859_2017_1563_MOESM6_ESM.pdf]

ProteoModlR

Paolo Cifani, Mojdeh Shakiba, Sagar Chhangawala and Alex Kentsis

October 24, 2016

Software documentation

Contents

1 Overview **1**
1.1 Applicability . . . 1
1.2 Functionalities . . . 2
1.3 Use alongside other computational tools . . . 2
1.4 Availability . . . 3
1.5 Software Requirements . . . 3

2 Input File **3**
2.1 Input File Format . . . 3
2.2 Simulated test datasets . . . 4

3 Workflow **4**
3.1 Quality control and Normalization . . . 4
3.2 Analysis . . . 6
3.3 Tutorial . . . 7
3.4 Suggested parameters . . . 8

1 Overview

ProteoModlR is an open-sourced R suite that facilitates extracting relative protein concentration and differential stoichiometry of post-translational chemical modifications from quantitative proteomics experiments.

1.1 Applicability

ProteoModlR processes quantitative proteomics data enabling researchers with minimal experience with quantitative mass spectrometry to assess differential activation of functional cellular processes. ProteoModlR is explicitly designed for datasets containing a set of synthetic peptided at equimolar concentration, but is compatible with other experimental design. ProteoModlR automates normalization to the equimolar peptides intensities to correct for variable ionization efficiency. Furthermore, it offers several modes of normalization to correct for different sources of error, and enables calculating differential protein expression and stoichiometry of post-tranlsational modification even from sparsely annotated datasets.

The computations are based on three assumptions:

1. A synthetic peptide reference is used, composed of peptides at equimolar concentration.
2. The peptides in assumption 1. are assumed to produce an MS signal linearly correlating to their amount, and their signal-response functions have slopes equal or close to one.
3. All or most of the variants of each peptides produced by chemical modification of a given protein (hereafter named chemoforms) are quantified.

ProteoModlR adopts a flexible annotation and allows for analysis of datasets from any experimental design that do not meet these assumptions. The software can be easily integrated with existing proteomics software, such as MaxQuant and Skyline, as well as with statistical and pathway analysis tools.

1.2 Functionalities

ProteoModlR performs three user-customizable functions: *quality control*, *normalization* and *analysis*. In *quality control* the data is checked for the presence of all specified columns in the correct order. Data is then filtered to remove peptides without any valid (i.e. >0) intensity measurement. *Quality control* classifies peptides based on their modification status with respect ot the user-specified PTM (e.g. "phosphorylation" or "acetylation"). For a given peptide sequence, if none of the chemoforms bears the modification of interest, then the peptide is and used for protein quantification (therefore labeled as Q). If, on the other hand, one or more chemoforms bear the modification of interest, then all the peptides are labeled as modified (M) or not modified (NM) depending on their annotated PTM status. The filtered and classified data is exported as a CSV file into the working directory (file name), along with a bar graph summarizing the number of peptides and proteins remaining after each step of quality control.

The second step is *normalization*, provided to correct differential ionization efficiency of different peptides in the datasets and other sources of technical variability. Here, the user can choose from four normalization options: normalization to isotopologue, normalization to equimolar isotopologue, normalization to internal reference peptide(s), and/or normalization to total ion current. If none are selected the data is returned unchanged. Otherwise, the normalized values replace the input intensities in the data. The normalized data is then exported as a CSV file into the working directory (file name).

The third step is *analysis*, where modification stoichiometry and peptide abundance are calculated. Depending on the user input on whether to restrict the analysis to exact calculations or to allow for approximate output (see section 3.2), the software selects the appropriate peptides based on the classification performed in quality control. ProteoModlR considers one modification per analysis. If positional information is provided, each modified site is considered separately. The analyzed data is exported as a CSV file into the working directory (file name).

ProteoModlR analyzes one user-specified PTM per analysis.

1.3 Use alongside other computational tools

ProteoModlR accepts as input any CSV formatted file with the appropriate columns. This type of file can be produced by a variety of softwares, such as Skyline and MaxQuant. The output of

ProteoModlR is also in a CSV format and is thus compatible with pathway analysis tools, such as Cytoscape, and with statistical analysis software for downstream analyses.

1.4 Availability

ProteoModlR and its documentation are available for download at
http://github.com/kentsisresearchgroup/ProteoModlR.

1.5 Software Requirements

ProteoModlR is an R suite and requires the following R packages, all of which are available through CRAN (https://cran.r-project.org): 'plyr','ggplot2','reshape2'.

2 Input File

2.1 Input File Format

The input file must contain 12 columns in the following order: `Protein`, `Peptide`, `Pathway`, `Modification`, `Gene.Name`, `Position`, `Protein.Name`, `Condition`, `PatientID`, `Label`, `Run`, `Intensity`, even if the content of the columns are left empty. Skyline users are encouraged to customize the output to fit this format. MaxQuant output (modification specific peptides table) can be easily re-formatted using external CSV editors.

1. `Protein`: This column stores the protein identifier, preferably as UniProt ID [www.uniprot.org]. If multiple identifiers are listed, the program takes the first 6 characters corresponding to the base of the first UniProt ID.
2. `Peptide`: this column stores the peptide sequence in single letter code.
3. `Pathway`: This column may store the pathway identifier to which each protein corresponds. This classification is entirely for post-analysis purposes and will simply follow the remainder of the data to the output. It can be used afterwards for pathway analysis in other software.
4. `Modification`: This column stores the modification(s) for to a given peptide. Use of UniMod nomenclature [www.unimod.org] is highly recommended. Note that the software tolerates minor variations in the modification identifiers. The use of dash in the modification ID (e.g. di-methyl instead of dimethyl) should be avoided. Peptides with no detected modification should be labeled "unmodified".
5. `Position`: This column may store the position within the peptide to which the modification in column 4 belongs. The content of this column is a text string.
6. `Gene.Name`: This column may store the gene name or gene ontology (GO) identifier for each protein.
7. `Protein.Name`: This column may store the protein name.
8. `Condition`: This column stores the experimental condition to which each peptide/protein corresponds. For instance, in a case-control study, this will be "healthy" or "diseased", while for a timepoint study this will be "T1", "T2, etc.
9. `PatientID`: This column may store the unique patient/sample identifier.

10. `Label`: This column stores the isotopologue labeling for each peptide (e.g. "H" for heavy-labeled and "L" for light-labeled isotopologues). For label-free datasets, label all entries as "L".

11. `Run`: This column stores the replicate ID for a given sample. For example if an experiment was performed in 3 technical replicates, each measurement may be labeled 1 to 3 in this column.

12. `Intensity`: This column stores the original, untransformed measure of abundance (i.e. MS intensity) as calculated using other software such as Skyline.

2.2 Simulated test datasets

ProteoModlR was tested using two sets of simulated datasets for the normalization and analysis modules respectively. Both sets were modeled on experimental intensity values obtained experimentally by targeted detection on a Thermo Orbitrap Fusion mass spectrometer of peptides from protein MEF2C, plus TUB1 and H1 as reference proteins. The datasets are available for download at http://github.com/kentsisresearchgroup/ProteoModlR.
Both dataset contains intensity values across three samples.
The *normalization* dataset simulates deviations from equimolarity introduced by different sources of technical and biological variability. The *analysis* dataset simulates missing measurements of different classes of peptides, for comparison with a fully annotated dataset.

3 Workflow

3.1 Quality control and Normalization

Once the data is made available in the working environment, the following code can be used to call both the *normalization* and the *quality control* functions with the desired inputs:

```
Normalize(data, iso.norm = "", equim.iso.norm = "", internal.norm = "", tot.current=
"", mod="")
```

Quality control checks for the presence and order of all required columns in the input files. If any chemoform of a given peptide bears the modification specified in `mod=""` (f.ex. `mod="phosphorylation"`) all peptides with the same sequence and containing the modification will be classified as *Modified* (M), while all peptides with the same sequence and not containing the modification will be classified as *Not-Modified* (NM). If none of the chemoforms of a peptide contains the specified modification, the peptide is classified *Quantification* (Q). After classification *Quality control* sums for each peptide the intensity of all *modified* and *not-modified* or *abundance* chemoforms.

`Normalize` takes in the data as a data frame. While the data file provided does not need to be exported from any specific program, the data should contain the 12 columns covered in Section 2. In addition to the data, the user can specify 5 arguments that determine the type of normalization to be performed (if any):

1. `equim.iso.norm` normalizes the intensity values in the input equalizing the intensity of all reference isotopologue, and preserving the L/H ratios. It takes a character string corresponding to the reference isotopologue to which the other isotopologue will be normalized (e.g.

`iso.norm = "H"`). The string must match the corresponding annotation in the "Label" column. This normalization first equalizes the intensities of all reference isotopologues (f.ex. "H") replacing the corresponding values with their median intensity calculated over the entire data frame. Normalized intensities (f.ex. light "L" peptides) are calculated as

$$Normalized\ Intensity\ nI_L^i = \frac{M_I^{DF} \times I_L}{I_H^i}$$

where I^i indicates the intensity of a peptide i either as light L or heavy H isotopologue, and M_I^{DF} indicates the median intensity calculated over the entire dataset.

2. `iso.norm` normalizes the intensity values in the input equalizing the intensity of isotopologue within each chemoform, and preserving the L/H ratios. It takes a character string corresponding to the reference isotopologue to which the other isotopologue will be normalized (e.g. `iso.norm = "H"`). The string must match the corresponding annotation in the "Label" column. This normalization equalizes, for each chemoform independently, the intensities of reference isotopologues (f.ex. "H"), preserving the L/H ratio for each peptide. Normalized intensities (f.ex. light "L" peptides) are calculated as

$$Normalized\ Intensity\ nI_L^i = \frac{M_I^{iH} \times I_L}{I_H^i}$$

where I^i indicates the intensity of a peptide i either in is light L or heavy H isotopologue, and M_I^{iH} indicates their median intensity for the heavy H peptides for chemoform i.

3. `internal.norm` normalizes the intensity values in the input equalizing the total ion current (TIC) across samples, and preserving the peptide/TIC ratios. Itnormalizes the intensity values in the input equalizing the intensity of reference peptides across samples, and preserving the reference/peptide ratios. It takes in a vector of character strings corresponding to the peptide sequence of the reference peptides to which other peptides are normalized (e.g. `internal.norm = "ATDVIVP"`). If more than one reference peptide is indicated (e.g. `internal.norm = c("ATDVIVP","AAATDVI")`), a geometric mean of the corresponding intensity values is taken. This normalization equalizes the intensities for the specified peptides in each sample. Normalized intensities for sample i are calculated as

$$Normalized\ Intensity\ nI^i = \frac{\overline{I_R^{eq}} \times I^i}{\overline{I_R^i}}$$

where $\overline{I_R^{eq}}$ indicates the (geometric mean) intensity of equalized reference peptide(s) R, $\overline{I_R^i}$ indicates the (geometric mean) intensity of reference peptide(s) R in sample i, and I^i indicates the non-normalized intensity in sample i.

4. `tot.current` takes in a boolean input indicating whether or not normalization to total ion current is to be made (e.g. `tot.current = T`). This normalization equalizes the sum of all intensities in each sample, used as a proxy for total ion current. Total Ion Current and normalized intensities for sample i are calculated as

$$Total\ Ion\ Current\ TIC^i = \Sigma I^i\text{; and}$$

$$Normalized\ Intensity\ nI^i = \frac{M_{TIC}\ \times\ I^i}{TIC^i}$$

where I^i indicates the intensity of a peptide i, M_{TIC} indicates the median TIC, and TIC^i indicates the TIC for sample i.
`mod` takes in a string corresponding to the protein modification of interest (e.g. `mod="phospho"`)

Lastly, any of the normalization options can be left unused by leaving the quotation empty or specifying the argument as false in the case of `tot.current`, but a modification must be selected for any analysis to be performed by the program.
The function `Normalize` will internally call the function for quality control and perform the filtering and classification needed for the next step. The data outputted from both the quality control step and the normalization step is saved in the working directory.

3.2 Analysis

The function `Analyze` calculates the approximate and/or exact site occupancy and/or abundance of the peptides:

```
Analyze(data, stoich="", abund="", ref.state="")
```

The first argument in the function call corresponds to the output of the function `Normalyze`. `stoich` takes in either `"Exact"` or `"Approximate"`, depending on the user's preference for calculating the site occupancy of peptides for which either calculations are possible. `abund` also takes in either `"Exact"` or `"Approximate"`, depending on the user's preference for exact or approximate abundance calculations. Relative abundance results are outputted as Δ-fold-change to facilitate downstream analyses.
Exact calculations are performed as follows:

$$Exact\ Abundance\ A^i = \frac{I_Q^i}{I_Q^{Ref}}\text{; and}$$

$$Exact\ Stoichiometry\ S^i = \frac{I_M^i}{I_{NM}^i + I_M^i};$$

where: I_Q^i indicates the intensity in sample i of a peptide which in the quality control module was annotated as "abundance" with respect to the user specified modification; I_Q^{Ref} indicates the intensity of the same peptide in the reference sampleRef ; I_x^iM indicates the intensity in sample i of a peptide x bearing the user specified modification; I_{xNM}^i indicates the intensity in sample i of a peptide x not bearing the user specified modification.

If some of the terms above are missing in one or more samples, and the user chooses to perform approximate analysis, the respective calculations are performed as follows:

$$Approximate\ Abundance\ a^i = \frac{I_{NM}^i + I_M^i}{I_{NM}^{Ref} + I_M^{Ref}};$$

(if no 'Q' peptide is available for a given protein) or

$$Approximate\ Stoichiometry\ s^i = \frac{I_M^i}{\bar{I_Q^i}}$$

(if no non-modified chemoform is available for a modified peptide).

where: $\bar{I_Q^i}$ indicates the mean intensity in sample$i$ of peptides which in the quality control module were annotated as 'Q' (Quantification) with respect to the user specified modification

Finally, results for both abundance and stoichiometry are expressed relative to the reference state/sample as Δ-fold change.

It is important to note that the decision as to whether exact or approximate calculations can be performed for a given peptide is made in the quality control stage, depending on the modification of a peptide and the existence of its unmodified form in the raw dataset. As such, if for instance exact abundance calculations are selected by the user, peptides for which only approximate abundance can be calculated are eliminated in the output of the function `Analyze`. The last argument in the `Analyze` function call is `ref.state`, which provides the user with the option to normalize to a reference condition (e.g. `ref.state="Disease"`).

3.3 Tutorial

- Set the working directory to that containing the dataset. This will be the folder where all the output data will be stored:

  For example: `setwd(Z:/Project/folder)`

- Install the package from GitHub using the following commands:

```
install.packages(devtools)
library(devtools)
install_github('kentsisresearchgroup/ProteomodlR')
library(ProteoModlR)
```

- The R package contains a test dataset that is based on the one generated by Ross et. al. (Immunity, 2016), shortened (randomly sampled set) for the purposes of testing to minimize the runtime. Once the package is installed, this dataset can be called by testData without the need to import (i.e. you can skip to step 3). When using a different dataset, the following command must be used"

```
test <- read.csv(inputfilename.csv)
```

- Call Normalization using the modification of interest and the type of normalization to be used.

  For example: `Normalize(data = test.Data, tot.current = T, mod = Phosphorylation)`

  This command will first run the data through the Quality Control module and then through the Normalization module. To perform no normalization, omit the entry for the normalization type:

`Normalize(data = test.Data, mod = Phosphorylation)`

QC can also be called directly using the following command:

`QC(data = testData, mod = Phosphorylation)`

- Once Quality Control/Normalization is completed successfully, a pdf file called Filtering_tracker.pdf is automatically exported into the working directory, showing a plot of the number of proteins and peptides remaining after each step of QC. In addition, the dataset exported from QC and from Normalize are saved to the working directory as Filtered_x.csv and Normalized_x.csv, where x is the time stamp.
- To calculate exact and/or approximate abundance and/or stoichiometry, Analysis module can be called using the following command: `Analyze(testData, mod, stoich = Approx", abund = Exact", ref.state = L)`
- The output of the Analysis module will be available in the working directory under the name that corresponds to the type of analysis performed, followed by the time stamp. In the example here, two CSV files would be created, approx_stoich_x.csv and exact_abund_x.csv, where x is the time stamp. These files can be easily exported into downstream graphing or statistical analysis software based on the users goals.
  Tips:
  - The premise, input, output and example call for each of the functions can be queried within R using the following commands:

```
?QC
?Normalize
?Analyze
```

3.4 Suggested parameters

While the design of ProteoModlR assumes the use of an equimolor set of isotopologue peptides, its flexible design is compatible with a variety of experimental work-flows. Applicability of specific Normalization and Analysis calculations must be carefully evaluated based for each study. The following suggested parameters for most common proteomics experimental designs are intended as an aid to new users and as example.

- Synthetic isotopologue reference peptides pool: All peptides of interest have a synthetic isotopically encoded counterpart, usually added to the sample at fixed concentration. Equimolar isotopologue normalization (`Normalize(data, iso.norm = "", equim.iso.norm = "H", internal.norm = "", tot.current= "", mod="")`) both corrects errors from differential ionization of peptides chemoforms and reduces technical variability. When applicable, equimolar isotopologue normalization enable the highest precision of downstream Analysis steps.
- Isotopically labeled reference proteome (SILAC, etc.): The majority of peptides have an isotopically encoded counterpart whose amount depends on cellular concentrations. Reference peptides are normally encoded with "heavy" isotopes. Isotopologue normalization (

`Normalize(data, iso.norm = "H", equim.iso.norm = "", internal.norm = "", tot.current= "", mod=""))` is recommended to reduce technical variability. *Caveat:* This normalization does not correct for differential ionization of different chemoforms, potentially resulting in decreased precision of stoichiometry calculations.

- Label free quantification: Normalization by TIC and by Reference Peptides may both be used, even in combination (`Normalize(data, iso.norm = "", equim.iso.norm = "", internal.norm = "PEPTIDE1,..., PEPTIDEn", tot.current= "T", mod=""))`, to reduce technical variability. The peptides used as internal reference should be unique to proteins whose cellular amount remains constant in all samples, such as house-keeping proteins (e.g. GAPDH, actin, etc). The user must preliminary ensure that the peptides chosen are detected in all samples, either by targeting them for detection (SRM, PRM, SIM) or because of their intrinsic observability (DDA, DIA). Normalization by TIC should only be used with comprehensive datasets (> 300 peptides) , with the majority of detected peptides ($> 80\%$) expected to be at similar cellular concentration in all samples. This conditions are usually verified in DDA and DIA experiments, but only rarely in targeted studies. *Caveat:* These normalizations do not correct for differential ionization of different chemoforms, potentially resulting in decreased precision of stoichiometry calculations.

- Enrichment of chemically modified peptides, such as those bearing phosphorylated (Me-IMAC,TiO_2, anti-pY antibodies), acetylated (anti-acetyl-K/R antibodies) and otherwise modified residues: peptide enrichment strategy alter the original PTM stoichiometry and may lead to stoichiometry over-estimation by ProteoModlR even when Exact calculations are applied. The use of equimolar isotopologue standard and of the corresponding normalization is mandatory to avoid such artifacts. Notably, stoichiometry overestimation is not an issue when enrichment is achieved for modified proteins before proteolysis.

- Study of multiply or sequentially modified peptides: PTM positional annotation of the input can be leveraged for functional analysis of modified peptides. In particular, the text string defining PTM position can be modified to reflect different modification patterns. For example, in the context of phosphorylation analysis ProteoModlR considers `Y2` and `Y4`, as distinct modifications, thus enabling the study of sequentially modified regulatory sites. Note that the positional annotation in the corresponding is considered as a text string. If no position is provided, ProteoModlR computes the global PTM stroichiometry, i.e. the fraction of all modified chemoforms bearing the specified PTM in any position.

- Data Dependent Acquisition (DDA): Unless deep sample fractionation is applied, datasets from DDA experiments are likely to contain sparsely annotated quantification and identification. Approximate calculations (`Analyze(data, stoich="Approximate", abund="Approximate", ref.state=""))` will then improve the comprehensiveness of abundance and stoichiometry annotation.

- Targeted Acquisition (SRM, PRM, SIM): Targeted methods are usually limited in comprehensiveness but optimized assays normally enable quantification of all peptides of interest. Exact calculations (`Analyze(data, stoich="Exact", abund="Exact", ref.state=""))` thus improve precision of the ProteoModlR output.

- Data Independent Acquisition (DIA): completeness of the annotation depends on the specific tools used, and fragmentation properties of the different chemoforms may selectively improve the quantification of modified or unmodified version of some peptides. Inspection of the annotations for specific proteins of interest is recommended to determine whether Approximate or Exact calculations are needed.
